# Experiences of treatment-resistant mental health conditions in primary care: a systematic review and thematic synthesis

**DOI:** 10.1186/s12875-022-01819-3

**Published:** 2022-08-16

**Authors:** Amelia Talbot, Charlotte Lee, Sara Ryan, Nia Roberts, Kamal R. Mahtani, Charlotte Albury

**Affiliations:** 1 Nuffield Department of Primary Health Care Sciences, University of Oxford, Radcliffe Primary Care Building, Radcliffe Observatory Quarter, Woodstock Rd, Oxford, OX2 6GG UK; 2 Faculty of Health and Education, Manchester Metropolitan University, Brooks Building, 53 Bonsall St, Hulme, Manchester, M15 6GX UK; 3 Bodleian Health Care Libraries: Cairns Library, John Radcliffe Hospital, Oxford, OX3 9DU UK

**Keywords:** Depression, General practice, Mental health, Primary care, Qualitative research, Systematic review, Thematic synthesis, Treatment resistance

## Abstract

**Background:**

Most adults fail to achieve remission from common mental health conditions based on pharmacological treatment in primary care alone. There is no data synthesising the reasons. This review addresses this gap through a systematic review and thematic synthesis to understand adults’ experiences using primary care for treatment-resistant mental health conditions (TRMHCs). We use the results to produce patient-driven recommendations for better support in primary care.

**Methods:**

Eight databases were searched from inception to December 2020 for qualitative studies reporting research on people’s experience with TRMHCs in primary care. We included the following common mental health conditions defined by NICE: anxiety, depression, panic disorder, post-traumatic stress, and obsessive-compulsive disorder. Two reviewers independently screened studies. Eligible studies were analysed using an aggregative thematic synthesis.

**Results:**

Eleven studies of 4456 were eligible. From these eleven studies, 4 descriptive themes were developed to describe a cycle of care that people with TRMHCs experienced in primary care. In the first stage, people preferred to self-manage their mental health and reported barriers that prevented them from seeing a GP (e.g., stigma). People felt it necessary to see their GP only when reaching a crisis point. In the second stage, people were usually prescribed antidepressants, but were sceptical about any benefits they had to their mental health. In the third stage, people self-managed their mental health (e.g., by adjusting antidepressant dosage). The fourth stage described the reoccurrence of mental health and need to see a GP again. The high-order theme, ‘breaking the cycle,’ described how this cycle could be broken (e.g., continuity of care).

**Conclusions:**

People with TRMHCs and GPs could break the cycle of care by having a conversation about what to do when antidepressants fail to work. This conversation could include replacing antidepressants with psychological interventions like talking therapy or mindfulness.

## Background

Mental health diagnoses are one of the leading causes of disease burden worldwide, with 10.7% or 792 million people diagnosed with any mental health condition [1, 2]. The global burden of disease study estimated that the disability-adjusted life-years due to mental health diagnoses increased from 80.88 million to 125.3 million between 1990 and 2019 [3]. The most common of these conditions are anxiety and depression, with European prevalence 6.38% [4]. These conditions are experienced by millions of people, with severe, disabling, and sometimes life-threatening symptoms [5].

Other common mental health conditions include panic disorder, post-traumatic stress, and obsessive-compulsive disorder [2, 6]. In England, one in four people experience a common mental health condition each year [6]. One in 50 people in the UK are also diagnosed with obsessive-compulsive disorder [7]. These statistics illustrate the need for increased mental health research, awareness, and treatment resources.

Psychological interventions like cognitive behavioural therapy are an evidence-based treatment option for common mental health conditions [8, 9]. However, average UK waiting times are twenty-eight to 90 days [10] compared to 139 days in Germany [11]. This means that common mental health conditions are often solely managed using pharmacological treatment in primary care [10]. The most common pharmacological treatments are antidepressants like fluoxetine, escitalopram, and sertraline, with 70.9 million people prescribed antidepressants in 2018 [12, 13].

While some antidepressants can effectively treat major depression [13], many people do not respond to this treatment [14]. This clinical response is known as treatment-resistance and is prevalent across common mental health conditions [15]. Cross-sectional data shows that 55% of British primary care users with depression report experiencing treatment-resistance [14]. The Sequenced Treatment Alternatives to Relieve Depression trial found that after 12–14 weeks of antidepressant usage, half of the participants did not experience a reduction of > 50% of depressive symptoms [16]. Other studies have found 10% of people with obsessive-compulsive disorder [17] and 33% of people with anxiety to be treatment-resistant [18].

British and American guidelines recommend referring people with potential treatment-resistance to secondary care services [19, 20]. However, there is a tension between these guidelines and clinical practice, as some people with treatment-resistant depression report trying twelve antidepressants and waiting 10 years between diagnosis and referral [21]. One specialist clinician in a treatment-resistant news article stated; “*there isn’t the capacity in secondary mental health teams to deal with this*” [21].

As part of the UK’s ‘Levelling Up’ agenda, primary care GPs are encouraged to refer anyone with common mental health conditions to Improving Access to Psychological Therapies (IAPTs) [22]. IAPTs provide psychotherapies and aim to see 75% of people within 6 weeks of referral [22]. The NHS website claims that IAPTs have “*transformed the treatment of adult anxiety disorders and depression in England*” [22]. However, some patients report waiting for up to 23 weeks (162 days) for an initial consultation [23]. Again, this means that treatment-resistant mental health conditions (TRMHCs) are mostly treated in primary care [21].

We found one study describing people’s experiences with treatment-resistant depression in primary care [14]. Participants in this study did not feel their depression was being managed adequately [14]. To our knowledge, studies like this have never been synthesised. A synthesis could consolidate evidence and identify ways to improve patient care. Reviews we found are quantitative and unrelated to primary care [24, 25]. These reviews may be less helpful to clinical practice given the aforementioned waiting times for IAPTs and secondary care. Other systematic reviews have examined how primary care can achieve better outcomes in common physical conditions like diabetes and high blood pressure [26–29]. However, the same logic has yet to apply to mental health conditions. This systematic qualitative review aimed to fill this gap to understand people’s experiences of primary care for the diagnosis and treatment of TRMHCs. We used thematic synthesis to summarise existing evidence, develop a high-order theme not observed by the original authors, and produce evidence-based implications for clinicians and policymakers [30].

## Methods

Our review is reported per the Enhancing Transparency in Reporting the Synthesis of Qualitative Research (ENTREQ) framework [31] and is registered online with Prospero: International Prospective Register of Systematic Reviews (ID: CRD42020216749) [32]. We consulted with three members of the public with lived experience of mental health conditions. They advised broadening the definition of TRMHCs from those defined by the National Institute of Health Excellence (NICE) (anxiety; depression, obsessive-compulsive disorder; panic disorder; post-traumatic stress disorder) [6] to include sub-conditions like bipolar, postnatal depression, and generalised anxiety disorder.

### Eligibility criteria

This systematic review synthesises qualitative research studies investigating adults aged > 18 with TRMHCs and their experiences of mental health provision in primary care. TRMHCs included anxiety; depression, obsessive-compulsive disorder; panic disorder; post-traumatic stress disorder [6]. Other inclusion criteria included:

- Any publication date or country of origin.
- Primary research published in peer-reviewed journals.
- Qualitative methods excluding methods that focused on language and social interaction (e.g., conversation analysis). This review focused on how people recount and interpret consultations rather than the linguistic detail of how social interaction is achieved [33].
- Studies sampling multiple populations where possible to extract data from people with TRMHCs.
- Studies where participants perceived themselves or were perceived by the original authors as having a TRMHCs, had been diagnosed, or matched definitions for treatment-resistance reported in a systematic review [15].
- Studies with a primary care context like accident and emergency, community services, general practice, and pharmacy. For studies with multiple contexts, only primary care data was analysed.
- Studies written in English to correspond to our language capacity. Studies translated by professionals were included to eliminate some English-language bias [34].

### Search strategy

The search strategy was created and run with NR, information specialist, and was based on **P**opulation, **I**nterest, and **Co**ntext criteria (PICo) [35]. This included: adults > 18 with TRMHCs (**P**), experiences (**I**), and primary care and treatment (**Co**). AT, qualitative researcher and lead author, inputted the search strategy into eight medical and social science electronic databases in December 2020. Databases included: CINAHL (EBSCOHost)[1982-present]; AMED (OvidSP)[1985-December 2020], Embase (OvidSP)[1974-present], MEDLINE (OvidSP)[1946-present], Global Health (OvidSP)[1973-December 2020] PsycINFO (OvidSP)[1806-present]; Sociological Abstracts (Proquest)[1952-present] and Google Scholar (https://scholar.google.com/). Included studies were citation searched. Searches were updated in November 2021, with no new studies found. The full search strategy can be found on the registered protocol [32].

### Study selection

Potentially eligible studies were imported into EndNote [36] with duplicates removed. Studies were then imported into Rayyan [37], a systematic review screening software, and previously undetected duplications were removed. Titles and abstracts were independently double screened by AT and CL for potentially eligible studies against the inclusion criteria [38]. The same two reviewers then used Rayyan to screen the full texts of remaining studies. All conflicts throughout the systematic review were resolved through discussion.

### Data extraction

Full-text PDFs of eligible studies were imported into NVivo12 for windows [39]. Methodological information was extracted to a piloted and modified Cochrane data extraction form by AT and checked by CL [40]. Features extracted to this tool included: authors; date of publication; focused condition; author definition of treatment-resistance; sample demographics and size; study setting; and method of data collection and analysis.

### Quality assessment

Studies were quality assessed independently by AT and CL using the Critical Appraisal Skills Programme (CASP), with a consensus reached through discussion. CASP [41] was used because it gives a good indication of the trustworthiness of the procedural aspects of studies [42]. Each ‘yes’ on the CASP awarded a study one point, ‘unclear’ half a point, and ‘no’ zero points. Congruent with other studies that have used CASP [43, 44], scores were evenly distributed, with 0–4 indicating low quality, 4.1–7 medium quality, and 7.1–10 high quality. We decided low quality was not a criterion for exclusion based on a previous systematic review which found that studies that did not score highly in the quality appraisal were not necessarily poor quality. Instead, some study aspects were not reported due to journal requirements or editorial decisions [45].

### Data synthesis

AT led a thematic synthesis using a modified version of Thomas and Harden’s approach [30]. Thomas and Harden’s [30] approach aims to go beyond data and develop high-order themes or ‘third-order interpretations.’ Our modified version involved systematic coding of data and the development of descriptive themes. We then created a high-order theme based on those descriptive themes. We chose a descriptive and interpretative approach because our review aimed to summarise existing evidence, rather than provide a purely theoretically driven review that ‘goes beyond’ existing knowledge [30, 46]. We incorporated interpretation to create potentially new, novel insights on treatment-resistant mental health conditions [30]. A similar thematic synthesis has been successfully applied in a systematic review of weight management [47]. Meta-ethnography was excluded because our research aim was not to ‘go beyond’ data [48].

The analysis had four steps:

1. Coding each line of data according to its context for each eligible study. This included abstracts, results/ findings, participant quotes, and discussions.
2. ‘Axial coding’ [49] to check that each line of data had been coded appropriately.
3. Grouping codes to develop descriptive themes using a mind map approach called the One Sheet of Paper (OSOP) method [50]. These themes were then written up.
4. Mapping the descriptive themes further to create a high-order theme not observed by the original authors. OSOPing involved reading through codes relevant to our research question and noting the different issues within these codes on a mind-map [50]. These notes were then linked together to describe core issues within the data [50]. This theme was then written up.

AT met with CA and SR several times throughout the analysis for alternative analytic insight. All team members agreed on the final themes. To ensure credibility (i.e., that the results are plausible), public contributors commented on whether the results resonated with their experiences [51]. All results were managed and analysed in NVivo12 [39].

## Results

**Fig. 1:**
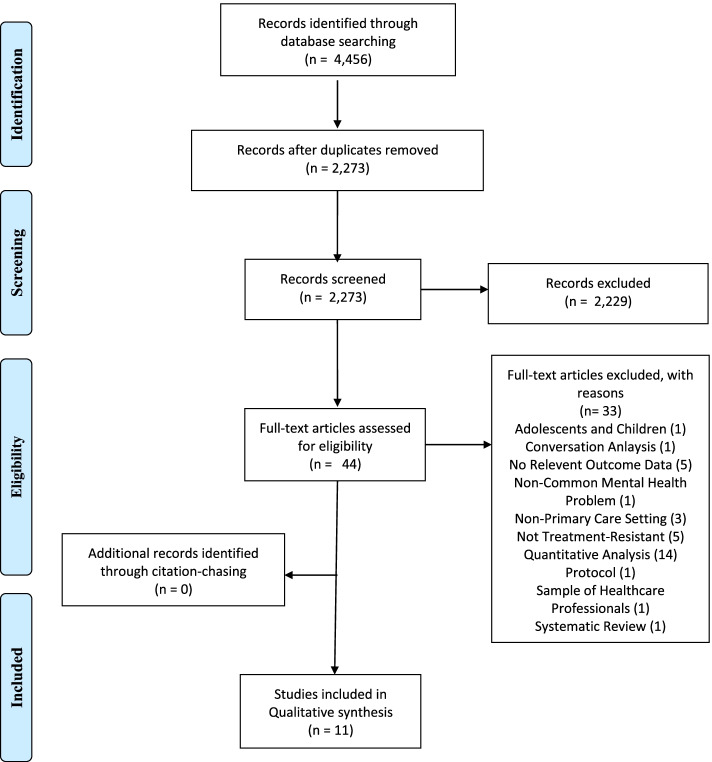
PRIMSA flow diagram and identification of studies

We found 4456 studies, of which 11 studies were eligible (Fig. 1). All but one study used interviews, with the other one using focus groups and participatory action. An additional three used focus groups alongside interviews [52–54]. The most common analytic approach was thematic [14, 54–58], alongside framework [14, 59], grounded theory [52, 53, 60], and narrative [61] methods. One study was conducted in Austria [58], one in Canada [61], one in Denmark [55], one in Malaysia [56], five in the UK [14, 53, 54, 59, 60], and two in the USA [52, 57]. Studies included African-American women [57], Latinos [52], Malay, Chinese, and Indian people [56], and adults > 61 years [61].

The contextual focus of four studies was primary care [14, 55, 59, 60]. The remaining studies were related to secondary care [60], or views of mental health conditions and mental health care generally [52, 54, 56–58, 61]. Authors classified treatment-resistance via depression inventories (psychometric tests), including the International Classification of Disease [62]. Treatment-resistance was also classed via diagnosis [52, 55, 57], evidence of recurrent depression [59, 61], continuous use of antidepressants [52, 55, 58, 60], seeking of secondary or tertiary care [57], and patient’s self-description [14]. No study sampled people with obsessive-compulsive disorder, panic disorder, or post-traumatic stress disorder. Table 1 summarises the characteristics of included studies.

**Table 1:** Characteristics of included studies

Author/s (Years)	Region, Country	Condition/s	Definition of Treatment-Resistance	Context	Data Collection Method	Participants	Demographics	Analysis Method
Buss, N. (2004) [55]	Southern Denmark	Depression/ Depressive Episode	Antidepressant prescription/ Depression Diagnosis/ Depression Inventory	Adherence to Antidepressants Primary Care	Longitudinal, Four Interviews	16	Age Range 29–38, Mean 50 10 Females, 6 Males	Thematic
Finucane, A. et al. (2006) [59]	Ayrshire, Scotland, UK	Recurrent Depression or Recurrent Depression and Anxiety	Depression Inventory, Current History of Recurrent Depression	Mindfulness, Primary Care	Interviews	13	Age Range 29–38, Mean 43 10 Females, 3 Males	Framework with Thematic Matrixes
Hansen, MC. et al. (2012) [52]	Los Angeles, California, USA	Diabetes, Major Depression	Depression Inventory	Pathways to Care	Interviews, Focus Groups	14 Interviews, 19 Participants in Focus Groups	All Spanish Speaking Latinos Mean Age 57 16 Females, 3 Males 12 Health Insurance, 7 No Insurance 18 Mexicans, 1 Peruvian	Grounded Theory
Ho, S. et al. (2017) [56]	Malaysia	Major Depression	Major Depression, Diagnosis, Depression Inventory, Taking Any Class of Oral Antidepressants > 6 Months	Outpatients from Psychiatric Hospital Barriers and Facilitators of Medication Adherence	Interviews	30	Age Range 27–59, Mean 45.2 15 Females, 15 Males 10 Malay, 10 Chinese, 10 Indian Hypertension 7, Diabetes 2, Asthma 3, Cardiovascular Disease 3, Other 4	Thematic
Johnston, O. et al. (2007) [60]	Southhampton, UK	Depression (including recurrent and persistent)	Self-Description	General Practice	Interviews	61	Age Range 18–83 44 Females, 17 Males 58 White, 1 Black Caribbean, 1 Pakistani, 1 Chinese 34 Paid Employment, 27 Unemployed	Grounded Theory
Kadam, UT. et al. (2001) [53]	UK	Anxiety, Depression	Depression Inventory	Views of Anxiety and Depression	Interviews, Focus Groups	27	Age Range 31–69, Mean 53 18 Females, 9 Males	Grounded Theory
Kessler, D. et al. (2018) [54]	UK	Treatment-Resistant Depression	Depression Inventory, Depression For > 6 Weeks and Taking Antidepressants	Medication Adherence	Interviews, Focus Groups	23	Age Raneg 27–76 15 Females, 8 Males	Thematic
Nicolaidis, C. et al. (2010) [57]	Portland, Oregon, USA	Major Depression	Depression Inventory	Racial Inequity in relation to healthcare	Focus Groups, Participatory Action	30	All African or African American All Women Age Range 19–53 Mean 36.2 All Low Income (67% Earn Less Than $16 k) Medicare 37%, No Insurance 40%	Thematic
Nussbaumer-Streit, B. et al., (2018) [58]	Vienna, Austria	Seasonal Effective Disorder	History of Seasonal Effective Disorder.	Prevention of Seasonal Depression	Interviews	10	Age Range 20–60 4 Females, 6 Males	Thematic
Reynolds, K. et al. (2020) [61]	Canada	Persistent Moderate to Severe Depression	Pursuing Outpatient Psychological Treatment at Tertiary Clinic.	Barriers to Care	Interviews	15	Age Range 61–86, Mean 72 11 Females, 4 Males	Narrative
Wiles, N. et al. (2018) [14]	Bristol, Exeter, Glasgow UK	Anxiety, Treatment-Resistant Depression	Depression Inventory Taking Antidepressants at An Adequate Dose for > 6 Weeks	Primary Care	Interviews	14	Age Range 18–75, Mean 45 10 Females, 4 Males 14 Secondary Diagnosis of Anxiety 6 > GCSE, 8 < A Levels 6 Paid Employment, 8 Not in Paid Employment 8 Homeowner, 6 Non-Homeowner	Thematic Framework Analysis

We judged all but one study to be high-quality using the CASP [41]. One study was judged to be medium quality because it did not clearly report its research design or recruitment methods or consider ethical issues [52]. Two studies were perceived as very high quality, scoring a 10 [55] and a 9.5 [58]. Other high-quality studies were perceived to be less clear on their recruitment strategies. For the full quality assessment results, see Table 2.

**Table 2:** Results of quality assessment using critical appraisal skills programme

Author (Year)	Statement of Aims	Appropriateness of Qualitative Methodology	Design	Recruitment	Relevance to Research Issue	Power Dynamics	Ethical Issues	Rigour of Analysis	Clear Statement of Findings	Value of Research	Score	Quality
Buss, N. (2004)	✓	✓	✓	✓	✓	✓	✓	✓	✓	✓	10	**High**
Finucane, A. et al. (2006) [59]	✓	✓	?	?	✓	✕	?	✓	✓	✓	7.5	**High**
Hansen, MC. et al. (2012) [52]	✓	✓	?	?	✓	✕	✕	✓	?	?	6	**Medium**
Ho, S. et al. (2017) [56]	✓	✓	✓	✓	✓	✕	?	✓	✓	✓	8.5	**High**
Johnston, O. et al. (2007) [60]	✓	✓	✓	✓	✓	✕	✓	✓	✓	✓	9	**High**
Kadam, UT. et al. (2001) [53]	✓	✓	✓	?	✓	?	?	?	✓	✓	8	**High**
Kessler, D. et al. (2018) [54]	✓	✓	✓	✓	✓	✕	✓	✓	✓	✓	9	**High**
Nicolaidis, C. et al. (2010) [57]	✓	✓	✓	✓	✓	?	✕	?	✓	✓	8	**High**
Nussbaumer-Streit, B. et al., (2018) [58]	✓	✓	✓	✓	✓	✓	?	✓	✓	✓	9.5	**High**
Reynolds, K. et al. (2020) [61]	✓	✓	✓	?	✓	✕	?	✓	✓	✓	8	**High**
Wiles, N. et al. (2018) [14]	?	✓	✓	?	✓	?	?	✓	?	✓	7.5	**High**

Key: ✓ = Clearly Met Criterion, One Point,? = Unclear If Criterion Met, Half A Point ✕ = Criterion Not Met, No Points

Score: 0–4 = Low Quality, 4.1–7 = Medium Quality, 7.1–10 = High Quality

### Descriptive themes summary

**Fig. 2:**
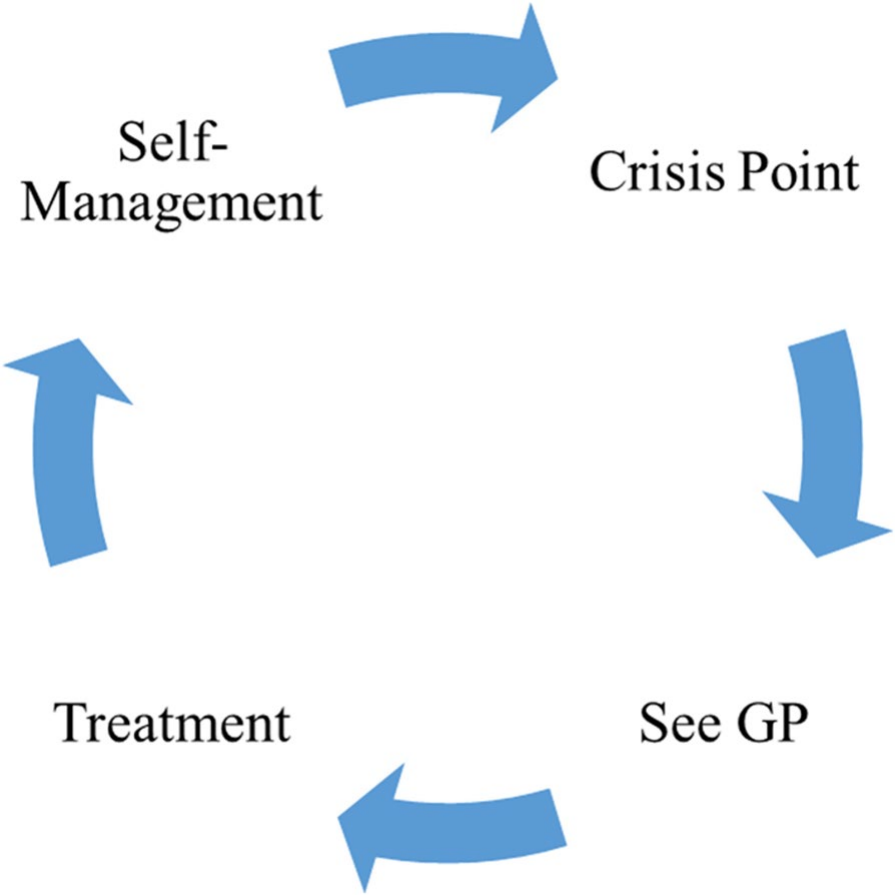
Cyclic care for trmhcs in primary care: visualisation of the descriptive themes

We developed four descriptive themes to reflect the content of included studies. These four themes describe a cyclic experience of primary care for people with TRMHCs. These stages are: barriers and crisis point; seeing a GP; treatment; and self-management (Fig. 2). We focus on people’s experiences with antidepressants because other medications were not mentioned in the primary studies. Primary quotations are presented to support our themes, and demographic information is included where provided in the original study. Table 3 shows the distribution of descriptive themes.

**Table 3:** Distribution of descriptive themes

Author (Year)	Descriptive Theme		Treatment	Self-Management	Total
	Barriers and Crisis Point	Seeing a GP			
**Buss, N. (2004)**	✓	✓	✓	✓	**4**
**Finucane, A. et al. (2006)** [59]			✓		**1**
**Hansen, MC. et al. (2012)** [52]	✓	✓	✓	✓	**4**
**Ho, S. et al. (2017)** [56]	✓	✓	✓	✓	**4**
**Johnston, O. et al. (2007)** [60]		✓	✓		**2**
**Kadam, UT. et al. (2001)** [53]	✓	✓	✓	✓	**4**
**Kessler, D. et al. (2018)** [54]	✓	✓	✓		**3**
**Nicolaidis, C. et al. (2010)** [57]	✓	✓	✓	✓	**4**
**Nussbaumer-Streit, B. et al. (2018)** [58]	✓	✓	✓		**3**
**Reynolds, K. et al. (2020)** [61]	✓	✓	✓		**3**
**Wiles, N. et al. (2018)** [14]		✓	✓	✓	**3**
**Total**	**8**	**10**	**11**	**6**	

### Stage one: barriers and crisis point

This stage refers to the barriers that prevented people from seeing a GP for their mental health and the point people understood their mental health as declining. One barrier included people’s preference to self-manage their mental health. Self-management activities included acupuncture, music therapy, exercise, prayer, aromatherapy, and dietary changes [53, 54, 56–58]. Other activities included late-night working, smoking, alcohol, and illicit drugs [14, 57]. Some participants described how they were engaging in these activities because they preferred to manage their mental health “*on their own*” and without the support of a GP [52, 53, 57]. These activities were not seen as a cure but as a form of respite [52, 53, 57].

It was evident that participants were self-managing their mental health because of perceived barriers to accessing primary care. For example, in five studies, participants mentioned that the perceived lack of emotional support from friends and family decreased their probability of booking an appointment with their GP [52, 53, 55–58, 61]. One participant spoke about how she hid her anxiety attacks from her family, who told her that her condition was “*stupid”* [53]. Another participant explained how her mother downplayed her depressive symptoms as “*growing pains*” that she could “*just walk off*” [57]. Participants with children felt that seeing a GP could cause unnecessary worry to other family members [52, 56, 61]:

“*You don’t want to overburden your children... they’re young, and they have little children and busy lives, and as a parent, you don’t want to be the needy one.”* (Female, 67 years, Persistent Moderate to Severe Depression) [61].

Across most studies, it was evident that stigma (including self-stigma) acted as a help-seeking barrier [14, 53, 55–57, 60, 61]. Participants commonly described their poor mental health as a “*trivial*” problem [53], that “*somebody’s worse off than we are, so we just got to deal*” [57], and that seeking GP support is “*an admission of failure”* [53]*.* Some participants were told by others to “*pull yourself together*” [53] and that seeing doctors for mental health was a sign of being “*loco (crazy)*” [52]. Many African-American women also spoke about how the “*strong black woman*” stereotype prevented them from seeking care [57]. These women also perceived prejudice within their healthcare system when it came to supporting black people’s mental health [57]:

“*My depression might not be like Suzie Ann’s depression, OK? Well, they’re going to call her name before they call my name. And they’re going to treat her just a little bit more different than me.*” (Female, African-American, Major Depression) [57]

Despite these perceived barriers, participants often felt that they had reached a crisis point where the symptoms were so severe that they had no choice but to seek medical help. Many participants used metaphors to communicate these experiences: “*anger ball*” [57], “*wanting to get out*” [52], “*I feel like I have something here [touching her chest], like a car*” [52], “*on edge*” [53], “*a volcano bursting”* [53], and *“a wall of pain*” [53]. Others were more direct with their descriptions, referencing the chronicity and severity of their symptoms [54]:

*“I had a lot of work stress going on as well, and it all got on top of me... I was massively overeating, oversleeping, permanent low mood, just generally unwell... At that point, I went to the doctor and said, ‘Look, this is what’s going on. I need some help with this”.* (Male, 52 years, Treatment-Resistant Depression) [54]

In contrast to earlier cases, some participants were motivated to seek medical support because of people in their personal network [52, 56, 61]. One participant mentioned seeking medical help after her eight-year-old daughter noticed her crying a lot [52]. Another participant was forced to go to hospital during a mental health crisis by her neighbour and was told by her sons, who had not realised how severe her depression was, to seek help [61]:

“*One night I phoned my neighbours and asked them to come over, cause I said I was feeling so awful. She came over, and said, “I think I’ll call an ambulance”. I said, “oh no, I don’t need an ambulance”. She said, "Well then, I’m taking you to the hospital". I was there until the next afternoon. Then my sons came, and they hadn’t realised what rough shape I was in, and they said I needed help.*” (Female, 70 years, Persistent Moderate to Severe Depression) [61]

### Stage two: seeing a GP

At the second stage, participants sought GP support for their mental health and appeared to feel confident that their GP could improve their depression [52–55, 61]. For example, one participant said that her GP had “*never let me down”* [53], and another said she had “a*bsolute faith*” in her GPs ability to treat her mental health [54]. For these participants, GPs were an accountable person who could facilitate discussions around accepting their mental health condition and deciding on possible treatments [52–55, 61]:

“*The doctor tells me. You have to accept your diabetes. You have to accept your high blood pressure. You have to accept....bad moods...you accept your problems, you have to accept your illness”... That is what I am trying to do, accept*” (Female, Major Depression, Diabetes) [52]

However, in seven studies, participants were disappointed by short consultations with their GP. They felt that short consultations did not provide enough opportunity to discuss their mental health [14, 52, 53, 56, 57, 60, 61], and in four studies, participants mentioned a lack of therapeutic continuity, which impacted on motivation to continue with treatment [52, 55, 56, 61]:

“*The doctor kept changing. If every time we see the same one, we would have more confidence in that doctor and will continue the treatment*.” (Male, Indian, Major Depression) [56]

Participants were further disappointed in the GPs advice because it did not meet their expectations around adjunctive care [55, 61]. In two studies, for example, participants felt their GP” *gave up*” because they did not offer follow-up appointments or suggest counselling or other non-medicinal therapies [55, 61]:

I: “*Why do you say that your general practitioner gave you up?”*

P: “*He just wrote the prescriptions, and then he was finished with me. He didn’t say that I should return; he didn't say that I should come for some counselling; he didn't say, “I’d like to keep track of you”. “You can come and get a renewed prescription, and we'll talk*.” (Female, Depression, Depressive Episodes) [55].

Participants in six studies felt that GPs did not have adequate mental health training and instead offered antidepressant treatment rather than providing a more detailed mental health assessment or opportunities to consider non-medicinal therapies [14, 55–58, 61]. Some participants felt like their consultations came off as robotic or like their GP was reading off a script [16]:

“*It’s as quick as they can get you out, write a script and out you go again.*” (Female, 51 years, Treatment-Resistant Depression) [14]

### Stage three: treatment

At stage three, participants engaged with antidepressant treatment. However, some also self-referred to psychotherapy [14, 52], secondary care services [14, 55], or were participating in a trial testing the acceptability of a mindfulness course prescribed in primary care [59]. The views of the mindfulness course were generally positive, with most describing improvements in their mental health, sleep, and reduced isolation due to practising in a group setting [59]. Only a few mentioned how the course did not fit with their schedule and disappointment that the course did not have the desired immediate impact on their mental health [59]:

“*I am able to deal with my emotions...I am not scared of things any more...I don’t want to turn about and walk away from things...I’ll take the time out to sit down and face up to it...”* (Recurrent Depression/ Recurrent Depression and Anxiety) [59]

Views of antidepressants were considerably more diverse. Participants in four studies reported a compromise between ambivalence towards the efficacy of antidepressants and hesitancy to discontinue due to possible relapse [14, 55, 56, 60]:

“*I didn’t wanna get involved in taking tablets for 6, 9, 12 months. I’m already 6 months into taking them now, which is longer than I thought... I thought, ‘Oh, I’ll get rid of it. I’ll be OK. I’ll have a few months, or I’ll have a couple of months off. I’ll be back to my normal self,’ but it hasn’t worked like that. Whether another antidepressant would help, I really don’t know*.” (Male, 55 years, Treatment-Resistant Depression) [54]

In this way, antidepressants were viewed as preventative against further mental health decline rather than treatment in itself [14, 52–54]. Participants felt antidepressants could support them to achieve a “*baseline*” level of functioning, and this could equip them to work on the social and psychological causes of their depression [14, 52–54]:

“*I’m on quite a low dose really, 20 mg of citalopram, and I think it was doing the job it needed to do … to get me to point where I could look at some issues.”* (Female, 39 years, Treatment-Resistant Depression) [54]

Subsequently, many participants felt antidepressants did not address the *‘true cause*’ of their depression [14, 53, 58, 59]. One participant described antidepressant discontinuation exacerbated the psychological causes of their depression (e.g. all-or-nothing thinking) [59] while other participants emphasised the importance of talking therapies to support more holistic recovery:

“*I just felt as if I wasn't in control anymore. They made me feel different [the antidepressants]. The same problems were there. So when I stop taking the tablets, I still had the emotional baggage and everything that I had stopped feeling when I started taking the pills... I've dealt with everything myself and at the end of the [mindfulness] course the feelings are still there, but I can deal with them so I would definitely feel that this* [the mindfulness] *is an alternative.*” (Recurrent Depression/ Recurrent Anxiety and Depression) [59]

### Stage four: self-management

At the fourth stage of the cycle, some participants stopped taking their antidepressants, mostly without the knowledge or support of a GP [14, 53, 55, 58, 60, 61]. Participants perceived a trade-off between the mental health benefits of antidepressants and the reported side-effects that affected other parts of their physical and mental health (worsened depressive symptoms, fatigue, affectless and apathy, sexual dysfunction, weight gain) [14, 53, 55, 58, 60, 61]. Under these circumstances, some participants followed advice from people in their personal networks who believed that long-term antidepressant treatment was not healthy [56, 57]:

“*My family members told me not to take this medicine [antidepressants]. They said it’s not good to take so many medications especially for long term....so I don’t take it*.” (Male, Chinese, Major Depression) [56]

Other participants experimented with antidepressants to counteract adverse side effects [14, 52, 53, 55–57]. Self-management included changing the dosage of the antidepressant, irregularly taking the antidepressants, and discontinuing antidepressants altogether [14, 52, 53, 55–57]. These self-management activities were thought to alleviate some of the perceived negative side-effects. Often participants had not told their GP about their experiments because they believed that they would not listen to their concerns about the side-effects and advise against it [14, 52, 53, 55–57]:

I: “*How come you took this decision [to self-medicate] without talking to the doctor?”*

P: “*It was probably because the doctor would be against it. I think I have an appointment in about a month from now. I thought that if I stopped them, I could see if it reduced my tiredness, and if there are no problems, then there is no reason to take them*.” (Depression, Depressive Episode) [55]

However, self-management of antidepressants was often unsuccessful and caused participants to relapse with their depression [14, 52, 53, 55–57]. Many of these participants eventually reached another crisis point and needed to see a GP for more support [14, 52, 53, 55–57]:

“*I tried to come off medication months ago, and I had a couple of little wobbles and stuff, so I went back on it*.” (Female, 26 years, Treatment-Resistant Depression) [14]

“*The only reason I still take them now is because a) I haven’t actually technically been told not, you know to come off them, and b) I just think it’s not a good idea to just suddenly stop them like I did last time.*” (Female, 26 years, Treatment-Resistant Depression) [14]

### High-order theme summary

**Fig. 3:**
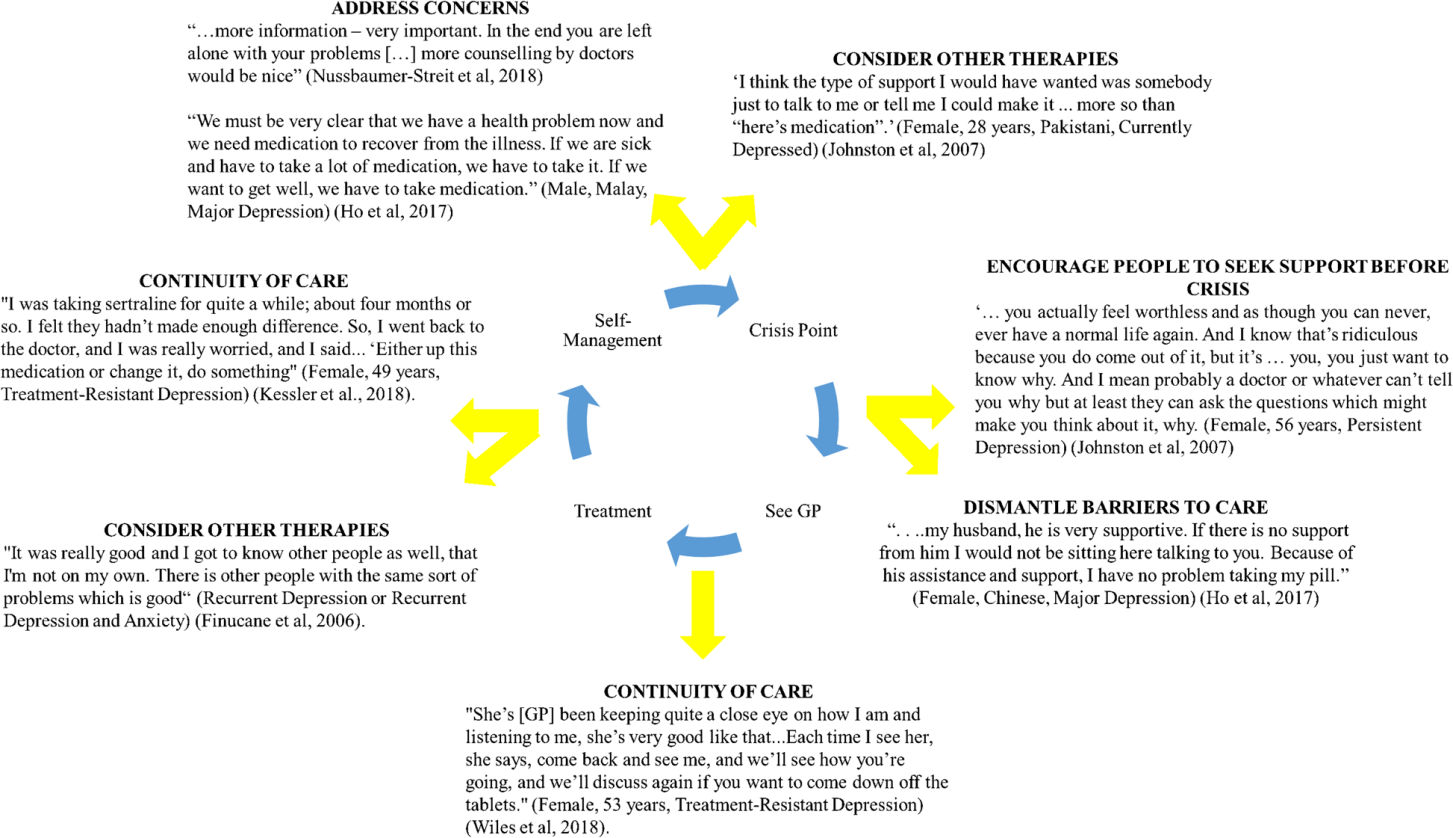
Creation of the high-order theme: breaking the cycle

So far, the results describe each stage of a mental health cycle that people with TRMHCs can experience in primary care. We mind-mapped the descriptive themes to develop a high-order theme [30, 50], not observed by the original authors, to explain how the cycle could be broken. We show how this high-order theme was created in Fig. 3.

### Breaking the cycle

Participants described opportunities to optimise the management of mental health conditions in primary care, which we interpreted as opportunities to break the cycle of care. In the first stage, support networks, including family and friends, encouraged participants to seek support from their GP [56–58, 61]. Seeking support early for a mental health condition helped people establish a relationship with their GP whereby they could ask questions about their depression, including treatment [56–58, 61]:

‘ … *you actually feel worthless and as though you can never, ever have a normal life again. And I know that’s ridiculous because you do come out of it, but it’s … you, you just want to know why. And I mean probably a doctor or whatever can’t tell you why but at least they can ask the questions which might make you think about it, why*”. (Female, 56 years, Persistent Depression) [60]

Having an established and continuous relationship with a GP allowed conversations about whether to change/or stop treatment helped participants instil confidence in their care plan and prevented a transition to stage four: self-management [14, 54–56, 58, 60]:

“*She’s [GP] been keeping quite a close eye on how I am and listening to me, she’s very good like that...Each time I see her, she says, come back and see me, and we’ll see how you’re going, and we’ll discuss again if you want to come down off the tablets.”* (Female, 53 years, Treatment-Resistant Depression) [14]

At all stages, participants appreciated shared-decision making. Participants wanted to discuss their depression and treatment options [14, 54–56, 58–60]. This could break the cycle by building people’s knowledge and confidence and subsequent engagement in the treatment option:

“*We must be very clear that we have a health problem now, and we need medication to recover from the illness. If we are sick and have to take a lot of medication, we have to take it. If we want to get well, we have to take medication*.” (Male, Malay, Major Depression) [56]

“*Well, since they treat me every six months … we hardly have talked, I only come and they look me over, and they say to me “where does it hurt, if it hurts”. They only prescribe me the medication and that is it*.” [52]

Generally, participants were aware of psychological therapies like counselling and mindfulness, and would have welcomed opportunities to talk with GPs about referrals to such services [14, 54–56, 58–60]:

“*I think it works* [counselling]*... Like you said, you don’t have to take the meds, you know, you don’t have to take the meds—just meet with your counsellor once a week for an hour*.” (Recurrent Depression/ Recurrent Anxiety and Depression) [59]

“*I think the type of support I would have wanted was somebody just to talk to me or tell me I could make it ... more so than “here’s medication*”. (Female, 28 years, Pakistani, Currently Depressed) [60]

Participants who continued with antidepressants wanted to see their GPs regularly about remission/ relapse and whether to change their antidepressants [14, 54–56, 58–60]. This could break the cycle by giving people regular opportunities to talk to the same GP about treatment effectiveness and jointly plan treatment changes:

“*I was taking sertraline for quite a while; about four months or so. I felt they hadn’t made enough difference. So, I went back to the doctor, and I was really worried, and I said... ‘Either up this medication or change it, do something*” (Female, 49 years, Treatment-Resistant Depression) [54]

## Discussion

### Statement of principal findings

This is, to our knowledge, the first qualitative systematic review to identify the cyclic use of primary care for TRMHCs. The cycle started with people showing resistance to seeking GP support for their mental health due to (self) stigma, a preference to self-manage, and a view that depression care could be influenced by racial prejudice. Nonetheless, participants felt and were advised by friends and family members to seek support when they reached a crisis point. Participants felt hopeful that their GPs could help them. Still, many felt disappointed when they were prescribed antidepressants without a more detailed mental health assessment, consideration of non-medicinal therapies, or offering follow-up appointments. Most participants felt ambivalent about how much their antidepressants were helping their mental health, were discouraged by the negative side-effects, and felt like they were not addressing the psychological cause of their depression. Many of these participants changed how they took their antidepressants, often without consulting their GP. For most participants, this was not successful, and their depression returned, which meant they had to see their GP again. However, there are several opportunities to break this cycle. These opportunities include promoting open dialogue, shared-decision making, and continuity of care.

### Strengths and limitations

This thematic synthesis has highlighted the cyclic care that people with TRMHCs can experience in primary care. While there was some diversity in the demographics of participants, with African-American women [57], Latinos [52], and Malay, Chinese, and Indian people [56] included, the majority of participants were white. The risk of poor mental health and unequal access to services are higher for people of Asian and Black ethnicities [63]. Therefore, the themes we report here may not fully capture the views of those who could most benefit from service reorientation. Most studies were conducted in the UK or other western countries (*n* = 11), which means that results may not be transferable to non-western contexts.

We used a recent systematic review to guide our definition of treatment resistance [15]. However, many of the studies required a significant level of interpretation because they did not clearly report their definition of treatment resistance. This presented a challenge and introduced the possibility that some studies may have been excluded inappropriately. This possibility was mitigated by using the conventional double-screening method [38], stringent inclusion and exclusion criteria, and discussion with the research team where there was conflict or where definitions were not initially clear.

### Comparisons with existing literature

A cross-sectional survey of the general UK population found that 35% of participants aged eighteen to twenty-five reported not seeking formal or informal mental health care [64]. The stigma associated with accessing medical support and talking about mental health was the most cited barrier to mental health help-seeking [64]. A World Health Organization survey also reported that a high percentage of people with mental health conditions do not seek or drop out of treatment, with 63.8% preferring to manage their mental health alone [65]. The stigma associated with seeking mental health support was identified in this review at stage one of the cycle. We also suggest that this stigma can prevent people from seeing a GP until they reach a crisis point.

At stage two, GPs were described as helping people accept their mental health condition and decision to receive treatment. We did not have the data to show how this could be done in practice. However, previous work on communication practice for delivering health behaviour change conversations in primary care demonstrates that collaborating with service-users via question-answer sequences can be well-received [66]. This supports findings within our high-order theme that participants wanted GPs to answer questions about the origin of their depression.

A qualitative article on primary care counsellors’ experiences of working with people with treatment-resistant depression found that counsellors worry about the omnipotence of depression [67]. However, counsellors find it difficult to help these people because of such omnipotence, resulting in less caring consultations [67]. While counsellors were not discussed in this review, many participants felt their GPs were not adequately equipped to treat their mental health. Statistics from Mind Charity show that 46% of trainee GPs in the UK undertake training placement in mental health settings [68]. GPs are also not required to undertake additional mental health training in their continuing professional development (CPD) [68]. This highlights GPs’ general low training in mental health and speaks to the importance of extending CPD criteria to include mental health.

In the high-order theme, we suggested that the cycle of care could be broken via continuity of care and patient-GP collaboration. Several studies have also shown the importance of continuity in relation to mental health care [69–71]. Bringer et al. [72] developed a five-step framework to support GPs with such continuity: Relationship, Timeliness, Mutuality, Choice, and Knowledge. The step ‘choice’ refers to how people prefer to have more than one support option available [72]. In our review, people also mentioned wanting a choice of a psychological intervention as adjacent to or instead of antidepressants. Other evidence-based interventions include GPs communicating about personal risk of depression, co-creating individualised psychosocial programmes [73], and internet-based CBT interventions [74].

### Implications for practice and policy

The findings demonstrate the importance of encouraging people to see their GP before they reach a mental health crisis. Two studies have shown that early diagnosis and treatment for depression (and many other mental health conditions) can potentially reduce the condition’s escalation [75, 76]. Dismantling structural barriers, including the stigma associated with mental health services, could be an important starting point for early diagnosis and treatment.

People with TRMHCs may benefit from a conversation with their GP about the efficacy, benefits/ risks of antidepressants, and what to do when antidepressants do not work. These conversations should be ongoing throughout the cycle to reduce the likelihood of people reaching stage four: self-management, and stage one: crisis point again. People may also benefit from their GPs talking about non-medicinal therapies like mindfulness which can improve depressive symptoms among people with recurrent depression [59]. This is in line with NICE guidance, which encourages GPs to decide with people with TRMHCs whether to replace their antidepressants with psychological interventions [19]. It is also congruent with the American Psychological Association’s recommendations for treating depression which state that psychological interventions should be recommended to people with mental health conditions [77].

Most studies were conducted in the UK, where routine screening for depression is not recommended [78]. A review of US evidence showed that routine screening for depression in the general adult population, including pregnant and postpartum women, helped identify depression early [79]. Early detection improved clinical outcomes [79]. We suggest that early screening may be equally beneficial elsewhere and reduce some of the identified barriers.

Our review demonstrates the importance of viewing mental health conditions as potentially long-term conditions. National initiatives toward monitoring long-term physical health conditions, like the UK Quality and Outcomes Framework, could be applied equally to mental health conditions [80]. Our review suggests that, in addition to routine monitoring of mental health conditions, continuity of care is vital for instilling patient self-efficacy in health care plans. This is supported by other evidence that continuity of care is essential to people with depression and can prevent hospitalisation, which can be distressing for patients [72, 81, 82]. Evidence has been distilled into the UK and American recommendations [8, 19, 77, 83, 84]. For example, the UK Community Mental Health Framework for Adults and Older Adults states that “*maximising continuity of care*” is key to “*delivering good mental health support*” [84, 85]. However, GPs are under various resource pressures, including growing workloads, a largely static workforce and the COVID19 pandemic [85, 86]. Therefore, careful consideration of the mental health needs of community populations is needed when allocating future resources to primary care.

Another option for supporting the management of long-term mental health conditions is the collaborative care model outlined by Fe et al. [87]. This model suggests that working with rather than for people may improve treatment outcomes [87]. This may include GPs engaging with patients’ ideas for treatment and management [87]. Evidence shows that collaboration can be more effective than conventional care models for mental health conditions [88].

### Implications for research

Two eligible mental health conditions, anxiety and depression, were captured in our synthesis. This evidences the need for research on treatment-resistant obsessive-compulsive disorder, panic disorder, and post-traumatic stress. Many people live with these conditions [17, 18], and without this knowledge, we do not know what current care looks like, nor do we understand how services could be improved. Researchers may find it challenging to recruit people with these conditions, given the lack of knowledge and consensus on what TRMHCs are. It may be beneficial, therefore, for researchers to engage with common-language definitions like “difficult to treat” [15]. Psychometric tests like the International Classification of Diseases [62] could then be used to assess the threshold at which a participant perceives themselves to be treatment-resistant.

The lack of understanding of treatment-resistance is a salient issue. More consensus between the public, medical, and academic communities is needed to increase the ability to research these conditions and ensure the transferability of findings. Researchers should be more transparent about how they define treatment-resistance; this could include reporting on individual depression inventory scores, number of medications tried, and duration on current medication.

## Conclusion

This systematic review and thematic synthesis has revealed that people with TRMHCs can experience cyclic care in primary care. This cycle consists of four stages: barriers and crisis point, seeing a GP, treatment, and self-management. The high-order theme showed that this cycle could be broken through continuity of care and open dialogue between GPs and people with TRMHCs. Future research could focus on mental health conditions such as treatment-resistant panic disorder, post-traumatic stress, and obsessive compulsive disorder.

## Data Availability

All datasets generated and/or analysed during the current study are available in the Open Science Framework, https://osf.io/465xm/.

## Abbreviations

CPD: Continuing professional development
GPs: General Practitioners. Also known as family doctors
NICE: National Institute of Care Excellence
PICo: Population, interest, context
TRMHCs: Treatment-resistant mental health conditions
IAPTs: Improving Access to Psychological Therapies
